# Green biologics: The algal chloroplast as a platform for making biopharmaceuticals

**DOI:** 10.1080/21655979.2017.1377867

**Published:** 2017-09-29

**Authors:** Henry N. Taunt, Laura Stoffels, Saul Purton

**Affiliations:** a Algenuity, Eden Laboratory, Stewartby, United Kingdom; b Algal Research Group, Institute of Structural and Molecular Biology, University College London, Gower Street, London, United Kingdom

**Keywords:** biopharmaceuticals, chlamydomonas, chloroplast, microalgae, synthetic biology

## Abstract

Most commercial production of recombinant pharmaceutical proteins involves the use of mammalian cell lines, *E. coli* or yeast as the expression host. However, recent work has demonstrated the potential of eukaryotic microalgae as platforms for light-driven synthesis of such proteins. Expression in the algal chloroplast is particularly attractive since this organelle contains a minimal genome suitable for rapid engineering using synthetic biology approaches; with transgenes precisely targeted to specific genomic loci and amenable to high-level, regulated and stable expression. Furthermore, proteins can be tightly contained and bio-encapsulated in the chloroplast allowing accumulation of proteins otherwise toxic to the host, and opening up possibilities for low-cost, oral delivery of biologics. In this commentary we illustrate the technology with recent examples of hormones, protein antibiotics and immunotoxins successfully produced in the algal chloroplast, and highlight possible future applications.

## Introduction

Biopharmaceuticals (protein biologics) is an industry estimated to be worth in excess of $140 billion^1^ and encompasses products such as monoclonal antibodies, immunotoxins, antigens, hormones, enzymes, clotting factors and bioactive peptides.^2^ These recombinant proteins are produced mainly using heterotrophic fermentation technologies with the biological platforms being either mammalian cell lines such as Chinese Hamster Ovary cells, or microorganisms such as bacteria or yeasts.^3^ Whilst these are highly advanced and successful technologies, there is a need for additional platforms that offer new opportunities for the production of therapeutic proteins. Emerging technologies include virus-mediate transient expression in insect cell lines^4^ or in tobacco plants,^5^ and stable expression in the chloroplasts of plants and algae.^6,7^

**Figure 1.**
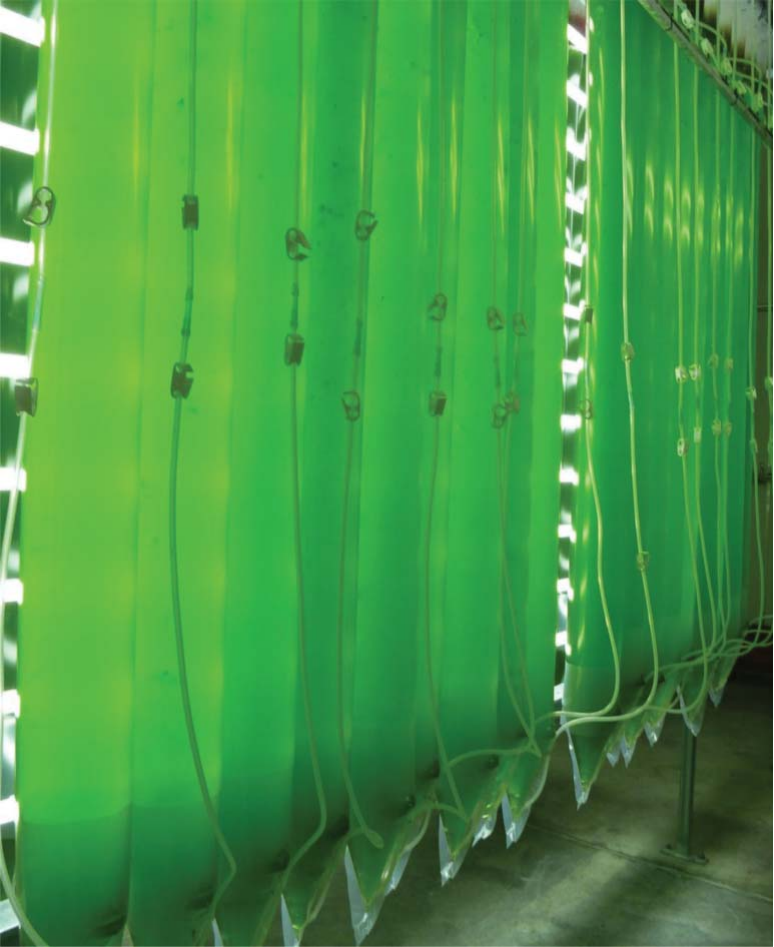
A low-cost, single-use photobioreactor system for commercial production of algal biomass. This ‘hanging bag’ system was developed by the Cawthron Institute, New Zealand for production of microalgae as aquaculture feed and for cultivation of *Haematococcus pluvialis* – a natural source of the high-value nutraceutical astaxanthin. We have successfully adapted the system for endolysin and vaccine production in *C. reinhardtii* (L. Stoffels, B. Parker and S. Purton, submitted). The 40 litre bags are optimally illuminated and sterile 5% CO_2_/95% air supplied at the base of each bag for phototrophic growth and for mixing. Both batch and continuous operation is possible. ©Supreme Health, New Zealand. Reproduced by permission of Supreme Health, New Zealand. Permission to reuse must be obtained from the rightsholder.

The use of unicellular algae as cell factories is particularly attractive as a low-cost, low-tech and sustainable approach, especially for countries lacking advanced fermentation infrastructures. As illustrated in Fig. 1, efficient production of algal biomass can be achieved in a cheap, sterile and disposable polythene tubing system that is easily scaled and managed. Each ∼40 litre ‘hanging bag’ is bubbled with CO_2_-enriched air and illuminated directly with sunlight, or indirectly using artificial lighting provided by LEDs powered by sunlight captured using photovoltaic devices. Whilst the latter adds to the capital costs, superior daily biomass productivities are obtained through 24 hour illumination using light of optimal intensity and wavelength, and tight control of the culture temperature. Cultivation of the algae uses a simple medium of basic nutrients, thereby keeping media costs as low as $0.002 per liter.^8^ Importantly, algal species grown commercially for the food ingredients and healthfood markets (e.g. species of *Chlorella, Dunaliella* and *Haematococcus*) already have GRAS (generally recognized as safe) status. The safety of these species offers the possibility of topical application of a biopharmaceutical such as an anti-microbial protein as a crude cell lysate (e.g. formulated into a cream or spray), and therefore avoiding costly investment in purification. Alternatively, it might be possible to use the whole algae for oral delivery (to animals, if not to humans) of vaccines, anti-microbials or hormones – with the dried cells exploited as a natural method of encapsulation and storage that overcomes the need for a costly cold chain, and the components of the algal cell possibly acting as an effective adjuvant.^9^

Recent surveys of the literature show that over 50 different biopharmaceuticals have been successfully produced in microalgae.^9,10^ Although production using nuclear genetic engineering is reported for several freshwater and marine species of eukaryotic microalgae, the majority of the research has focused instead on chloroplast engineering using the freshwater green alga *Chlamydomonas reinhardtii*. Insertion of transgenes into the small chloroplast genome rather than the nuclear genome offers several clear advantages: not least the ability to do precise and predictable ‘DNA surgery’ in which transgenes are integrated into specific, neutral loci within the genome via homologous recombination, and stable, high-level, stable expression is readily achieved.^11^ Furthermore, protein folding and disulphide bond formation occurs readily in the chloroplast allowing the correct assembly of complex therapeutic proteins with multiple domains or multiple subunits, as discussed below. Finally, the chloroplast compartment can serve as a safe sub-cellular site for hyper-accumulation of recombinant protein without affecting the biology of the rest of the cell.^12^ The growing interest in exploitation of the algal chloroplast is now driving the development of synthetic biology tools by ourselves and other groups that allow a rapid and efficient pipeline for design and production of engineered strains. Below we highlight this technology and give three examples of applications in the field of biopharmaceuticals.

### The *C. reinhardtii* chloroplast as an emerging synbio platform

**Figure 2.**
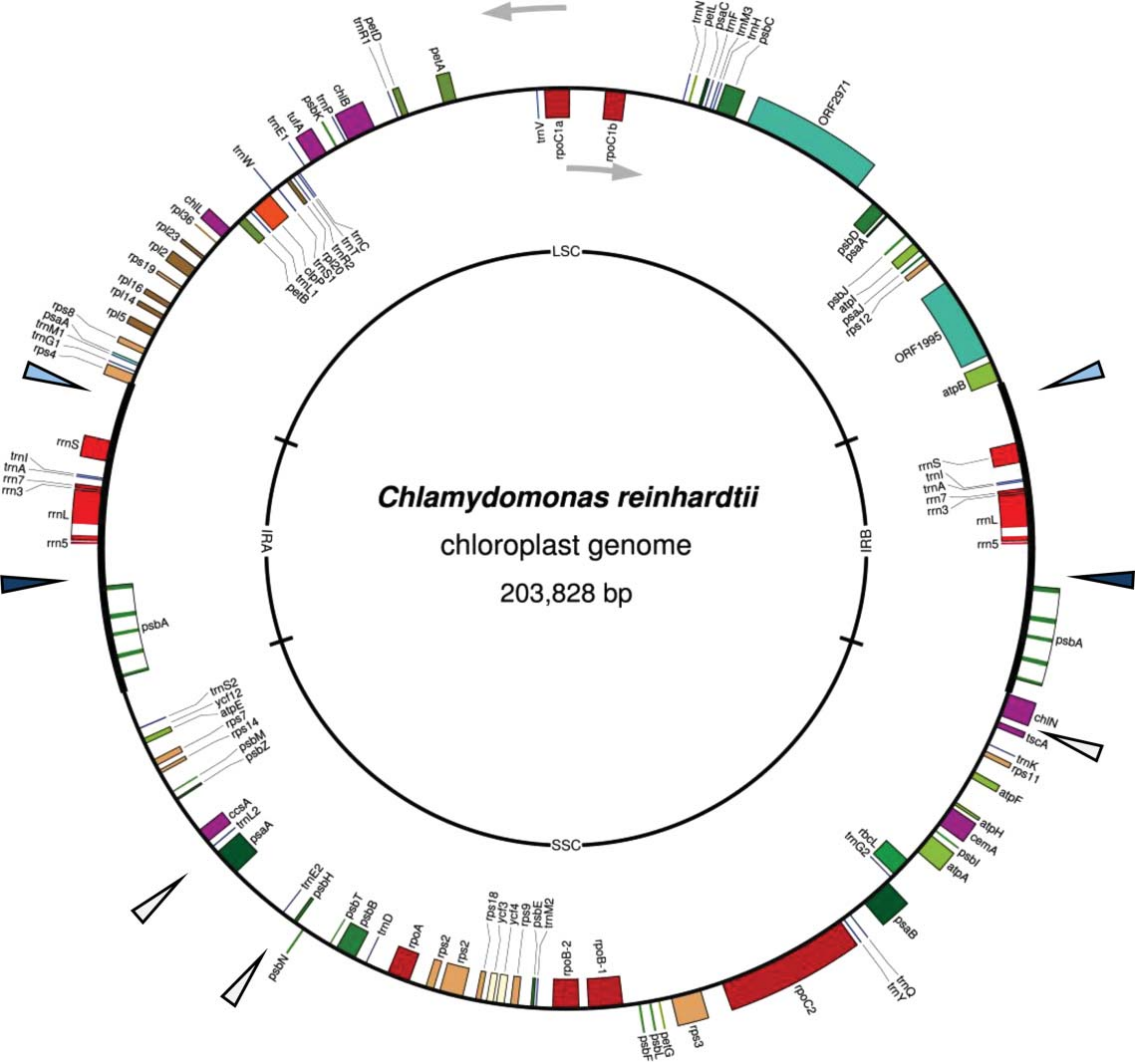
The chloroplast genome of *Chlamydomonas reinhardtii*. Generated from Genbank entry BK000554 using OGDRAW (ogdraw.mpimp-golm.mpg.de). Genes are coloured according to function (e.g. photosystem II genes in dark green), with genes transcribed anticlockwise on the outer side of the circle; those transcribed clockwise on the inner side. Examples of verified neutral sites for transgene insertion are indicated by arrowheads, with those within the inverted repeat (IR) regions that therefore give rise to two transgene copies per genome shown in light or dark blue.

Chloroplast genomes (or ‘plastomes’) are polyploid circular molecules possessing 100–200 genes, with most encoding core components of the photosynthetic apparatus or the organelle's transcription-translation machinery (Fig. 2). Gene structure and expression is essentially prokaryotic in nature, reflecting the evolution of the chloroplast from a cyanobacterial ancestor. Hence, genes are often arranged as operons, transcribed by a eubacterial-type RNA polymerase and the mRNA translated on 70S ribosomes.^13^ Chloroplast transformation was first achieved using *C. reinhardtii* whereby a photosynthetic mutant carrying a chloroplast gene deletion was restored to phototrophy by microparticle bombardment with a plasmid carrying the wild-type gene. Molecular analysis showed that the mutant locus had been repaired through efficient homologous recombination (HR) between sequences on the plastome and the introduced DNA. Since then, *C. reinhardtii* has been used extensively as a laboratory model for reverse-genetic studies of chloroplast gene expression and photosynthetic function, with specific gene knockouts or site-directed changes introduced into the plastome through HR-mediated engineering.^14^

**Figure 3.**
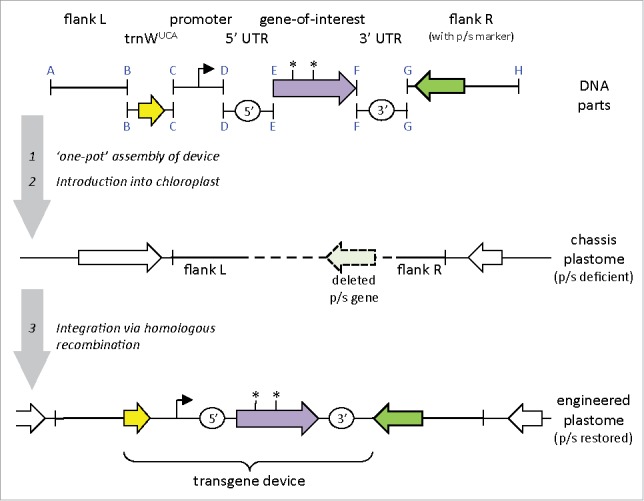
A synbio strategy for creating marker-free transgenic lines that also incorporate a biocontainment feature. Standardised DNA parts are assembled in order using Golden Gate to create the transgene device, with left (L) and right (R) flanking plastome elements (shown as bold lines) added for homologous recombination in the chloroplast. One element carries a wild-type copy of an essential photosynthetic (p/s) gene allowing phototrophic selection in the recipient chassis that lacks this gene. The synthetic gene-of-interest is codon-optimised and fused to promoter and untranslated region (UTR) parts. Biocontainment can be incorporated into the transgene by replacing one or more tryptophan codons with the UGA stop codon (*), thereby preventing function transfer of the gene to other microorganisms. Correct translation in the chloroplast is achieved by inclusion of a part carrying trnW^UCA^. This gene encodes an orthogonal variant of the chloroplast's tryptophan tRNA that recognises UGA.

More recently, the focus has shifted to biotechnological applications and the development of the *C. reinhardtii* chloroplast as a protein factory through the addition of novel genes into the plastome to make valuable recombinant products.^11^ Improvements in the transformation technology have helped to advance this field and we now are beginning to see the application of synthetic biology (synbio) principles. These include gene design *in silico* using dedicated codon optimization software and validated *cis* elements such as promoters and untranslated regions.^15,16,17^ Building the designed constructs *in vitro* is then aided by rapid assembly of standardized DNA parts using methods such as Golden Gate^18^ that ensure the ‘one-step’ assembly of multiple parts in the correct order and orientation (Fig. 3). Accompanying this are methods for large-scale refactoring of the plastome and for regulating the expression of the transgenes.^19,20,21^ Finally, the development of strategies for ‘marker-free’ generation of transgenic lines that avoid the use of antibiotic resistance markers,^22^ and a technique for bio-containment of the transgene through codon reassignment^23^ will help to address regulatory issues and public concerns regarding commercial cultivation of transgenic microalgae. Further details of these tools are given in Fig. 3.

### Three case studies: Human growth hormone, endolysins and an immunotoxin

Human growth hormone (hGH) is a 22 kDa protein that is produced naturally in the pituitary gland. Deficiency of the hormone results in growth defects, but can be successfully treated by administration of recombinant hGH.^24^ As the only post-translational steps required for biological activity are removal of the N-terminal methionine and formation of two intrapeptide disulphide bonds, then recombinant production is feasible using a prokaryotic host. *E. coli* is currently the preferred platform, although correct folding and bond formation requires export into the periplasm.^25^ However, the demand for recombinant hGH is huge and growing, with a predicted global market of $4.5 billion by 2022, thereby justifying the exploration of alternative production platforms including chloroplasts. Recent work by our group has demonstrated that functional hGH can be produced in the *C. reinhardtii* chloroplast by expression of a codon-optimized synthetic gene fused to the promoter and 5′ untranslated region of the endogenous *psaA* gene.^22^ Yields of hGH in the transformant were approximately 500 µg per liter of culture, so there is a need to increase this significantly before we can compete with bacterial platforms. Nevertheless, biological activity could be demonstrated even in crude cell lysates using a standard assay where addition of the lysate specifically stimulated growth of a rat lymphoma cell line. This work highlights the potential of the algal chloroplast as a future platform for making simple biopharmaceuticals such as hGH, insulin and bioactive peptides.

In a second study from our group, the production of endolysins in the *C. reinhardtii* chloroplast was investigated.^26^ Endolysins are antibacterial proteins produced during bacteriophage infection that digest the bacterial cell wall for phage progeny release at the end of the lytic cycle. These enzymes typically show a high degree of specificity for the target bacterium of the phage. Furthermore, the emergence of resistance to endolysins appears to be extremely rare. Consequently, endolysins have potential as protein antibiotics, with recombinant forms shown to be highly effective when added to bacterial cultures or biofilms.^27^ The chloroplast is a particularly attractive platform for recombinant production since it mimics the prokaryotic environment where endolysins are produced naturally, but unlike bacterial hosts, it lacks any peptidoglycan cell wall that might be compromised during over-expression of an endolysin gene. Using the same strategy as for hGH production, two different endolysins – Pal (36 kDa) and Cpl1 (40 kDa) – that target the major human pathogen *Streptococcus pneumoniae* were successfully produced in *C. reinhardtii*. Each enzyme showed a high lytic activity against cultures of *S. pneumoniae* even when presented as crude cell lysates, suggesting that the algal platform could be used for simple, low-cost formulations of anti-bacterial creams or sprays targeting topical bacterial infections, or infections of the nasal pharynx.

**Figure 4.**
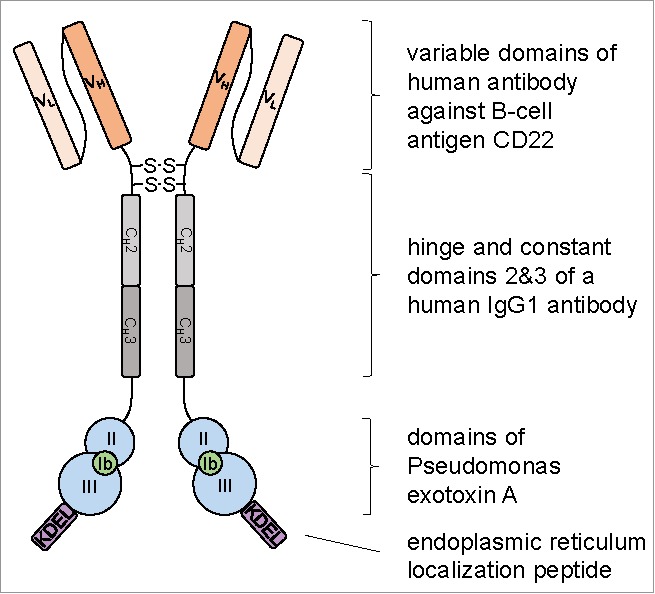
Illustration of a designer immunotoxin produced in the *C. reinhardtii* chloroplast showing the multi-domain structure. The human CD22-scFv domain was fused to the hinge and constant domains of a human IgG1 and to exotoxin A from *Pseudomonas aeruginosa* lacking domain 1a. This created an immunotoxin that formed a homodimer through disulphide bonds between the hinge regions. Redrawn from^12^.

The third case study illustrates a possible niche for the algal chloroplast platform that addresses issues encountered with existing prokaryotic and eukaryotic hosts. Tran et al.^12^ investigated the synthesis of immunotoxins in the *C. reinhardtii* chloroplast. These chimeric proteins are targeted therapeutics that have applications in cancer treatment, and consist of an antibody domain for binding to the target cell and a cytotoxic enzyme that inhibits proliferation of the cell. As shown in Fig. 4, the immunotoxin is a complex multi-domain protein that requires correct folding and disulphide bond formation to generate the active homodimer. Production of such proteins within bacterial hosts is challenging because these expression platforms often fail to fold proteins with multiple domains efficiently and are unable to form disulphide bonds. Conversely, production of such cytotoxic proteins in eukaryotic hosts such as CHO cells or yeast is not feasible because of the lethal effect of the toxin on the cytosolic translation apparatus. The work of Tran et al.^12^ demonstrates that the algal chloroplast not only possesses the machinery necessary to fold and assemble complex eukaryotic proteins, but that the 70S ribosomes are unaffected by the toxic protein and the organelle is able to contain the protein preventing any inhibitory effect on the host's cytosolic ribosomes. The chloroplast therefore offers an attractive platform for efficient production of these highly complex therapeutics.

### Resources and future applications

Advances in the genetic engineering of the *C. reinhardtii* plastome, in particular the application of synbio strategies, have simplified and accelerated the process of creating designer strains expressing therapeutic proteins. In our lab, we have sought to develop a simple, low-cost pipeline that can readily be adopted by other groups, including those in developing countries. Chassis strains and DNA parts are available through the Chlamydomonas Resource Center (www.chlamycollection.org) and our software for codon optimization is free to download (github.com/khai-/CUO). Our chloroplast transformation protocol simply involves agitation of a cell/DNA suspension in the presence of glass beads, rather than the use of expensive microparticle bombardment equipment, and we have developed a simple PCR-based method for confirming transgene insertion and homoplasmy of the plastome.^22^ Our on-going work on scale-up using the hanging bag system shows that this is a cost-effective and easily manageable cultivation method; and biomass productivity could be further improved through optimization of key parameters such as light, CO_2_ delivery, mixing and media composition.^28^

Currently, recombinant protein yields are low (typically 0.5-5% of total soluble protein) compared to established microbial platforms, but better understanding of chloroplast gene regulation and the use of orthogonal mechanisms to induce and drive transgene expression,^29^ should lead to marked improvements. Indeed, recombinant protein levels achieved in chloroplasts of tobacco have been reported as high as 70% of total soluble protein.^30^ In addition, protein productivity could be improved through genetic enhancement (“domestication”) of the chassis strains to improve their performance in photobioreactors.^31^ Alternatively, the chloroplast engineering technology could eventually be transferred to faster-growing and more robust native species of green algae such as *Chlorella* that are better suited to intensive commercial cultivation.^32^

Possible applications of the algal chloroplast platform extend beyond human therapeutics, and are particularly attractive where the cost of production and storage are key issues. For example, microalgae are a natural part of the diet for insect larvae, juvenile shellfish, fish fry, etc. Consequently, engineered *C. reinhardtii* strains have been proposed for oral delivery of toxins to insect pests such as mosquitoes,^33,34^ or delivery of vaccines and growth hormones to farmed fish and shellfish.^35^ Similarly, the GRAS status and nutritional value of various green algal species opens up the possibility of “functionalized feed” for poultry and livestock whereby dried algae formulated into the feed also contains beneficial vaccines, anti-microbials or dietary enzymes.^36^

To date, no biopharmaceuticals produced in microalgae has been approved for commercial production and only a handful have been tested in animal experiments. Significant further research and development of microalgal platforms is therefore required. However, conventional production of protein biologics is expensive and technically demanding – requiring capital-intensive fermentation facilities, and costly downstream processing, cold storage and transportation, and sterile delivery methods. To meet the future needs of the global population, alternative low-tech, low-cost and sustainable production systems such as microalgae must be considered.
